# Alternatively Spliced Tissue Factor Is Not Sufficient for Embryonic Development

**DOI:** 10.1371/journal.pone.0097793

**Published:** 2014-05-30

**Authors:** Susanna H. M. Sluka, Alexander Akhmedov, Johannes Vogel, Dusten Unruh, Vladimir Y. Bogdanov, Giovanni G. Camici, Thomas F. Lüscher, Wolfram Ruf, Felix C. Tanner

**Affiliations:** 1 Cardiovascular Research, Institute of Physiology, University of Zurich, Zurich, Switzerland; 2 Center for Integrative Human Physiology, University of Zurich, Zurich, Switzerland; 3 Institute of Veterinary Physiology, University of Zurich, Zurich, Switzerland; 4 Division of Hematology/Oncology, Department of Internal Medicine, University of Cincinnati College of Medicine, Cincinnati, Ohio, United States of America; 5 Cardiology, Cardiovascular Center, University Hospital Zurich, Zurich, Switzerland; 6 Department of Immunology and Microbial Science, The Scripps Research Institute, La Jolla, California, United States of America; Maastricht University Medical Center, Netherlands

## Abstract

Tissue factor (TF) triggers blood coagulation and is translated from two mRNA splice isoforms, encoding membrane-anchored full-length TF (flTF) and soluble alternatively-spliced TF (asTF). The complete knockout of TF in mice causes embryonic lethality associated with failure of the yolk sac vasculature. Although asTF plays roles in postnatal angiogenesis, it is unknown whether it activates coagulation sufficiently or makes previously unrecognized contributions to sustaining integrity of embryonic yolk sac vessels. Using gene knock-in into the mouse TF locus, homozygous asTF knock-in (asTFKI) mice, which express murine asTF in the absence of flTF, exhibited embryonic lethality between day 9.5 and 10.5. Day 9.5 homozygous asTFKI embryos expressed asTF protein, but no procoagulant activity was detectable in a plasma clotting assay. Although the α-smooth-muscle-actin positive mesodermal layer as well as blood islands developed similarly in day 8.5 wild-type or homozygous asTFKI embryos, erythrocytes were progressively lost from disintegrating yolk sac vessels of asTFKI embryos by day 10.5. These data show that in the absence of flTF, asTF expressed during embryonic development has no measurable procoagulant activity, does not support embryonic vessel stability by non-coagulant mechanisms, and fails to maintain a functional vasculature and embryonic survival.

## Introduction

Tissue factor (TF) triggers blood coagulation and supports non-hemostatic processes through cell signaling. The TF transcript can undergo alternative splicing of exon 4 to 6, which creates a soluble protein isoform termed alternatively-spliced TF (asTF) with a unique C-terminus that lacks the transmembrane and intracellular domains of full-length TF (flTF) [1]. Although of different lengths, the C-termini of human and mouse asTF show appreciable sequence homology within the first 40 amino acids [2].

It is controversial whether asTF exhibits procoagulant activity [1], [3], [4], [5]. Although asTF lacks important residues that in flTF interact with macromolecular substrate, the majority of residues interacting with FVII(a) are present in flTF and asTF [6]. Recombinant murine asTF exhibits minimal, phospholipid-dependent cofactor activity [2]; nevertheless, procoagulant activity of murine asTF has not been characterized in in vivo in the absence of flTF.

TF is crucial for embryonic development, since TF-deficient embryos fail to sustain a functional yolk sac vasculature and display embryonic wasting between days 9.5–10.5 [7]. A transgene expressing human flTF at ∼1% of normal murine TF activity is sufficient to rescue embryonic lethality of knockout mice lacking murine TF [8] without restoring normal postnatal hemostatic function [9]. Mice deficient in FV, prothrombin, and protease activated receptor 1 (PAR1) display a very similar defect in yolk sac vasculature development and embryonic lethality with a lower penetrance of 50–60% [10], [11], [12].

In postnatal angiogenesis, asTF has non-hemostatic roles, increasing endothelial cell migration and adhesion by signaling via integrins [13]. It is not known whether the increased lethality in TF knockout embryos relative to other knockouts of other factors of the thrombin/PAR1 pathway results from unrecognized roles of asTF in embryonic vascular development.

In this study, we investigated whether, in the absence of flTF, expression of asTF during embryonic development yields sufficient procoagulant activity to improve integrity of yolk sac vasculature and/or supports vessel stability by non-coagulant mechanisms [13].

## Methods

### Ethics Statement

All animal experiments were approved be the cantonal veterinary office of Zurich, Switzerland (Protocols 62/2009 and 75/2012).

### Generation of asTFKI Mice

Murine asTF open reading frame was obtained by PCR amplification of mouse aortic mRNA and inserted into KpnI/NotI restriction sites of a LNTK vector (PolyGene AG, Ruemlang, Switzerland) located 5′ to a Neomycin resistance gene flanked by two loxP sites (floxed neo). A 3 kb genomic sequence 5′ of the first exon of the TF gene and a 5.2 kb fragment located 3′ of the last exon of the TF gene were used as the left and right homology arms, respectively. A diphtheria toxin alpha chain minigene (dtα) under control of the DNA polymerase II promoter served as a negative selection marker; dtα was inserted at the 3′-end into the targeting vector. The targeting vector was linearized at its 3′-end prior to electroporation. Embryonic stem cell transfection (129P2/OlaHsd), screening, cre recombinase-mediated Neomycin excision, and blastocyst injection were conducted by PolyGene AG (Ruemlang, Switzerland).

Chimeric mice were bred with C57BL/6J mice for germline transmission of the mutated allele. The mouse colony was maintained by breeding of heterozygous animals on a mixed 129P2/OlaHsd-C57BL/6J background. In four heterozygous asTFKI mice, the open reading frame of the introduced construct was sequenced and revealed 100% sequence identity with the murine asTF cDNA transcript (www.ensembl.org: ENSMUST00000090417). For embryo analyses, heterozygous asTFKI mice (F2–F4 generation) were bred and females were checked for vaginal plugs every morning. The day of the plug was considered to be E0.5.

### Transcript and Protein Expression Analyses

Uteri were dissected at E9.5. Embryos were snap frozen and yolk sacs were used for genotyping. Total RNA was extracted from embryos using TRIzol Reagent (Molecular Research Center) according to the manufacturer’s instructions. 2 µg of RNA was used to synthesize cDNA using Ready-To-Go You-Prime First-Strand Beads (GE Healthcare) and random hexamer primers (TaKaRa). Expression levels of the TF isoforms were measured using TaqMan expression assays (Applied Biosystems) with a probe spanning the exon 4 to 5 boundary for flTF (Mm00438856_m1) and a probe spanning the exon 4 to 6 boundary for asTF (Mm01316952_m1). Eukaryotic 18S rRNA (4333760T) was used for normalization of expression. Amplification efficiencies were measured with serial 10-fold dilutions and were identical for flTF and asTF. Relative expression levels represent multiples of ten of 2^(Ct[flTF]-Ct[S18])^ or 2^(Ct[asTF]-Ct[S18])^. Total protein was extracted by manual grinding of the embryos in a lysis buffer containing 50 mM octylglucoside, 50 mM Tris, 150 mM NaCl, 10 µg/µl aprotinin, and 10 µg/µl leupeptin pH 7.4. Lysates were precipitated with −20°C acetone overnight and resuspended in non-reducing sample buffer with careful sonication. Samples were separated by 10% SDS-PAGE and transferred to a PVDF membrane (Immobilion-FL, Millipore). The membrane was stained with polyclonal rabbit anti-mouse TF antibody (R8084) [14], polyclonal rabbit anti-mouse asTF antibody [2], and anti-human GAPDH (cross-reactive to mouse) mouse monoclonal (MAB374, Millipore) primary antibodies, followed by staining with goat anti-rabbit 800CW and donkey anti-mouse 680 LT (LI-COR Biosciences) secondary antibodies. Staining was visualized on an Odyssey imager and quantified using the corresponding application software (version 3.0; both LI-COR Biosciences).

### TF Activity Assay

Procoagulant activity was determined in whole embryos using a plasma clotting assay [15]. Uteri were dissected at E9.5, embryos were snap frozen, and yolk sacs were used for genotyping. Embryos were lysed by manual grinding in HEPES-saline containing 0.02% sodium azide (HBS) After total protein quantification with a Bradford assay (Bio-Rad), embryo lysates were diluted in HBS containing 1 mg/ml BSA and 50 µM phospholipid vesicles (70% phosphatidylcholine (PC) and 30% phosphatidylserine (PS), Avanti polar lipids) to measure TF activity using a plasma clotting assay. Human and mouse citrated plasma was mixed 9∶1 (50 µl) in order to provide mouse factor VII for optimal mouse TF procoagulant activity and prewarmed together with the sample (50 µl) or a reference (recombinant lipidated human TF, American Diagnostica). Clotting times were measured after addition of 25 mM CaCl_2_ (50 µl). Procoagulant activities were calculated using a standard curve prepared with serial dilutions of recombinant human TF and normalized to the total protein concentration of the sample.

### Embryo Preparation for Histology

At E8.5, 9.5, or 10.5, uteri were fixed with 4% formalin in PBS for at least 4 days. Using a razor blade, individual implantation sites were separated and each implantation site was halved transversely to the uterus walls. From one half, a piece of embryonic tissue was taken and washed with PBS before genotyping followed by dissection of the yolk sac for en face evaluation. The other half was embedded in paraffin and sectioned to perform H&E or immunohistochemical staining of smooth muscle α-actin (Sigma, F3777). The primary FITC labeled mouse anti α-SMA antibody was detected with a rabbit anti-FITC antibody. The latter was detected with a biotinylated anti-rabbit antibody marked with a HRP labeled streptavidin-biotin-complex. The diaminobenzidine chromogen developed brown color reaction. Immunohistochemical staining using the anti-mouse asTF polyclonal antibody or rabbit IgG isotype control (Jackson Immunoresearch) was performed as described [2].

Yolk sac erythrocytes or α-SMA^+^-cells were counted manually and normalized to the cross-sectional length of the yolk sac which was determined using the AnalySIS FIVE software (Quality Report).

### Statistical Analysis

Data are shown as mean ± SEM. χ^2^ analysis was used to compare genotype distributions. Unpaired Student’s t-test was performed for comparison of two groups, one-way ANOVA with Bonferroni posttest was used to compare three groups and two-way ANOVA with Bonferroni posttest was used to compare genotypes at different timepoints. p values<0.05 were considered significant.

## Results

### Generation of asTF Knock-in Mice

**Figure 1:**
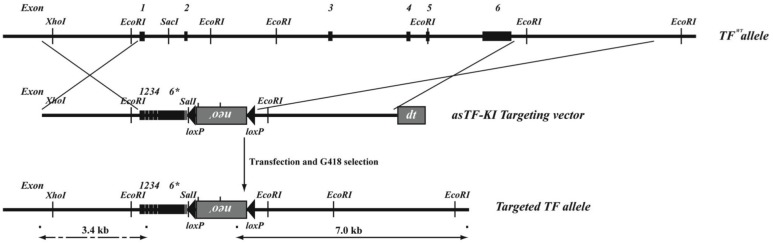
Targeting of the TF gene. The murine TF allele was targeted with a replacement-type vector containing the murine asTF open reading frame flanked by 3 kb 5′ and 5.2 kb 3′ homology arms. Diphtheria toxin (dt) was used for negative selection. After homologous recombination into 129P2/OlaHsd embryonic stem cells, a loxP flanked neomycin resistance cassette was removed by transfection with a Cre expression plasmid.

To study the function of asTF in the absence of flTF in vivo, an asTF knock-in (asTFKI) mouse was generated by replacing the TF gene with the murine asTF coding sequence under the control of the murine TF(*F3*) promoter (Fig. 1A). This novel genetic model enabled investigation of asTF function in the physiological setting of embryonic development.

### Homozygous asTFKI Embryos Die after Day E9.5

**Figure 2:**
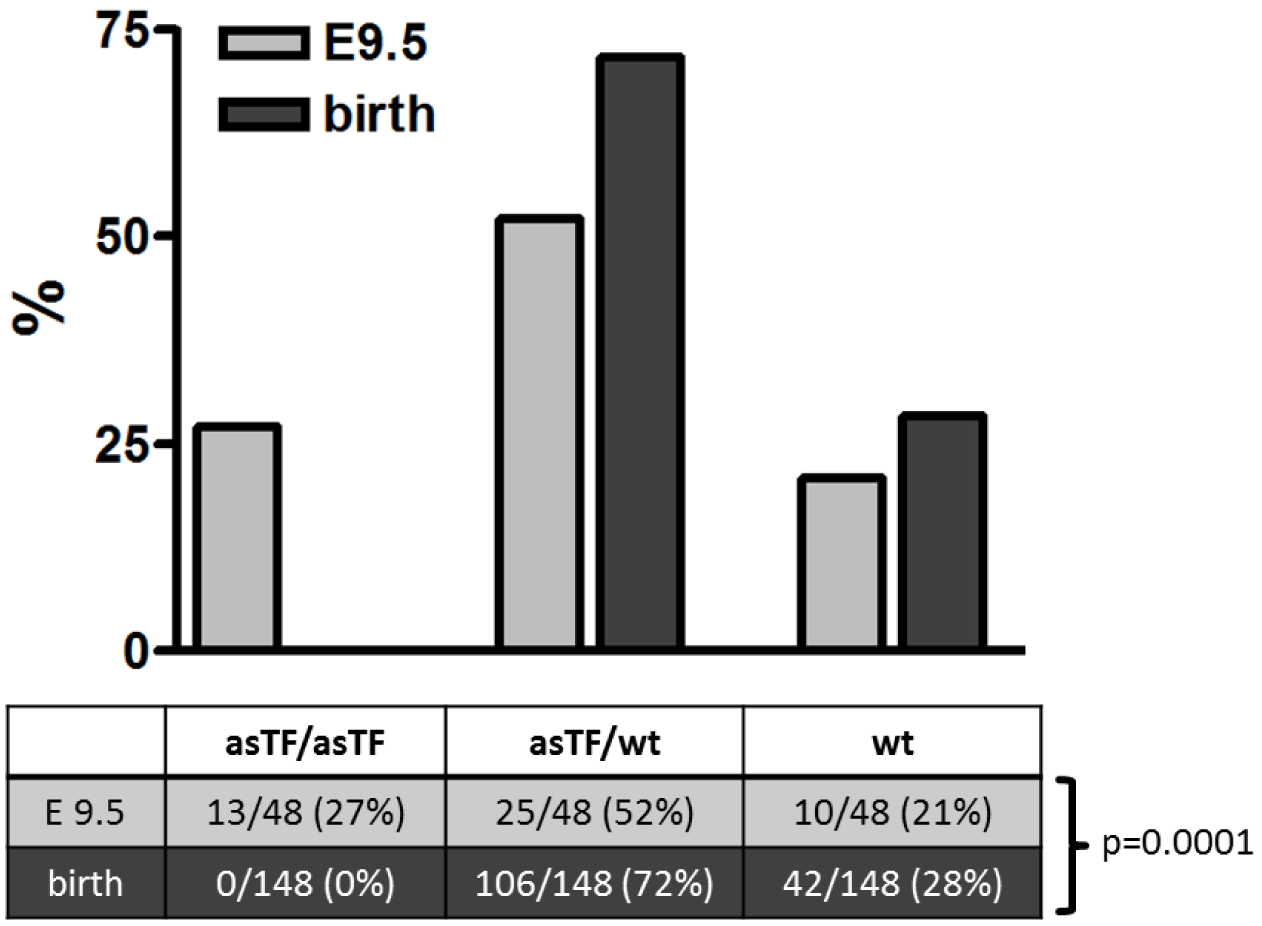
Genotype distribution on E9.5 and birth. Homozygous asTFKI embryos die after developmental day E9.5. Genotype distribution of offspring from heterozygous breeding pairs at birth (black bars) differs from that at embryonic day 9.5 (grey bars). At day 9.5, embryonic genotypes are distributed in a Mendelian manner, while no more homozygous asTFKI pups can be identified at birth (p = 0.0001, χ2-test).

We genotyped 148 offspring from 3 different heterozygous asTFKI/wtTF breeding pairs and found no homozygous asTFKI, 106 heterozygous asTFKI/wtTF (72%), and 42 (28%) wt animals (Fig. 2). Deletion of the TF gene in mice leads to embryonic lethality between embryonic day 9.5 and 10.5 [7]. To determine whether homozygous asTFKI embryos develop to this stage, 48 E9.5 embryos were genotyped. 13 homozygous asTFKI (27%), 25 heterozygous asTFKI/wtTF (52%), and 10 wt (21%) embryos were identified, demonstrating normal Mendelian segregation significantly different from the genotype distribution found at birth (p = 0.0001) (Fig. 2). Thus, asTFKI embryos die during development after day 9.5.

### Expression of TF Splice Isoforms in E9.5 Embryos

**Figure 3:**
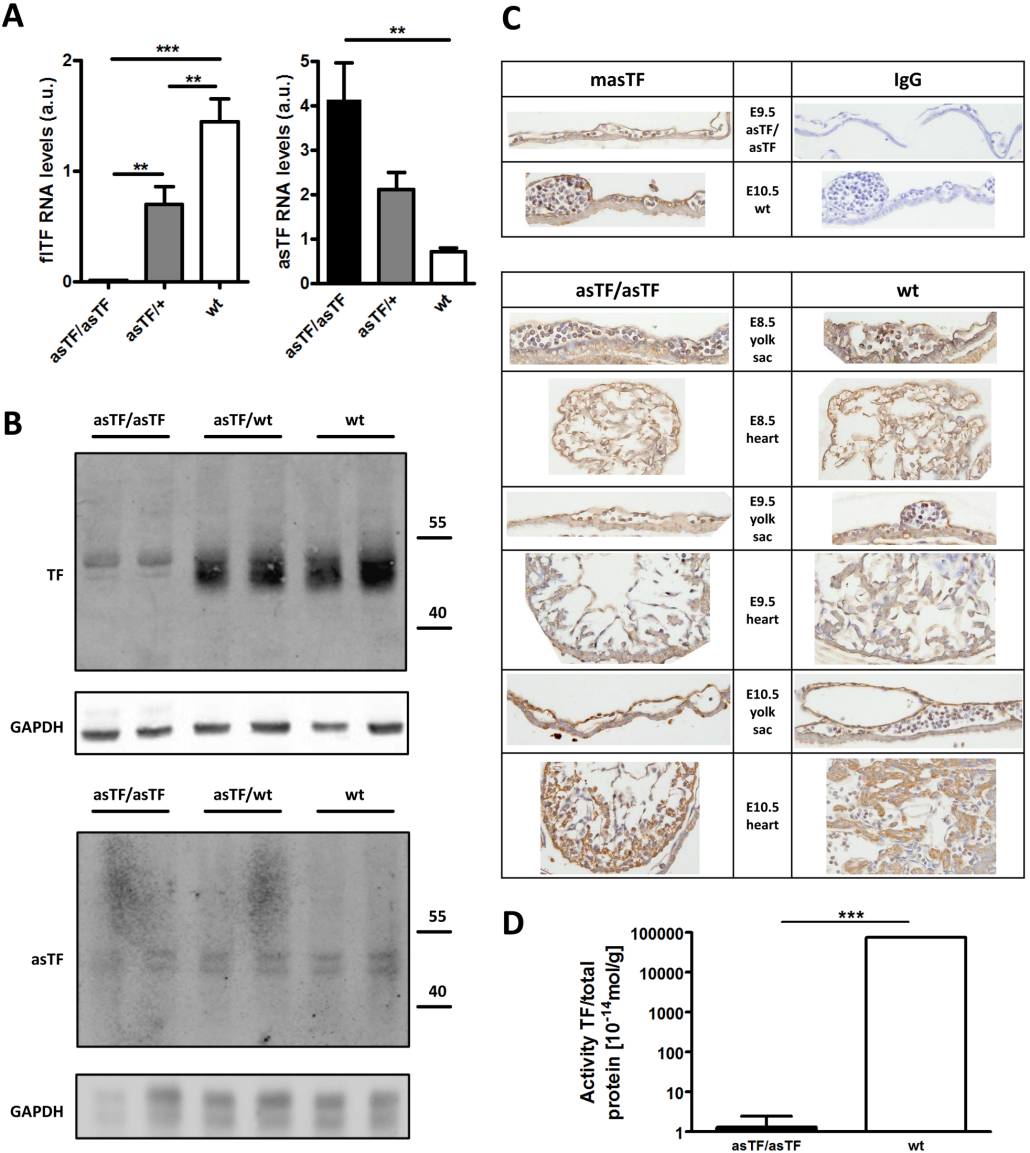
Expression and activity of asTF. (A) RNA levels of asTF and flTF were measured in E9.5 whole embryos and normalized to S18 expression, N = 5–7, ***P<0.001, **P<0.01 (B) Protein expression of total TF (western blot probed with a rabbit anti-mouse TF polyclonal antibody), asTF (western blot probed with a rabbit anti-mouse asTF polyclonal antibody), and GAPDH in E9.5 whole embryos. Intensity of normalized total TF staining relative to wt (100%) was 74% in asTFKI/wtTF embryos and 19% in asTFKI/asTFKI embryos. Expression of asTF was comparable between homozygous asTFKI, heterozygous asTFKI/wtTF and wt embryos (C) Mouse asTF and rabbit IgG control immunohistochemistry on E9.5 homozygous asTFKI and E10.5 wt yolk sac. Mouse asTF immunohistochemistry on E8.5 to E10.5 extraembryonic (yolk sac) and embryonic (heart) tissue of homozygous asTFKI and wt embryos. (D) TF activity of whole embryo lysates was measured with a plasma clotting assay and normalized to total protein. N = 5, ***P<0.001.

Wt embryos expressed both flTF and asTF mRNA. The mRNA expression of flTF decreased by 52% in heterozygous asTFKI/wtTF embryos (p<0.01) and was undetectable in homozygous asTFKI embryos (p<0.001 vs. wt). mRNA expression of asTF was 3-fold higher in heterozygous asTFKI/wtTF (p>0.05) and 5.9-fold higher in homozygous asTFKI embryos (p<0.01) relative to wt embryos, which is a magnitude of expression expected from the switch to a single splice variant expressed by one or two alleles, respectively (Fig. 3A). Using an antibody reactive with both forms of murine TF [14], we observed partial overlap of flTF and asTF bands on western blots (Fig. 3B). Homozygous asTFKI embryos displayed expression of a more homogenous TF protein, with a predominant higher apparent molecular weight species also seen in wt and heterozygous asTFKI/wtTF embryos. Since three extracellular N-linked glycosylation sites of flTF are preserved in asTF, the observed apparent molecular weights of the splice isoforms most likely result from size differences in the protein as well as carbohydrate structures [2]. Quantification of TF protein levels revealed that homozygous asTFKI embryos expressed 19% of total TF detected in wt embryos, whereas heterozygous embryos expressed 76% of wt levels. Western blotting using an antibody selectively reactive with murine asTF [2] showed comparable expression of asTF in homozygous asTFKI, heterozygous asTFKI/wtTF, and wt embryos, indicating that the introduced asTFKI allele did not cause a major overexpression relative to endogenous asTF. Immunohistochemical staining using the anti-mouse asTF antibody [2] confirmed, in a semi-quantitative manner, comparable expression of asTF in embryonic (heart) and extraembryonic (yolk sac) tissues in homozygous asTFKI and wt embryos, and throughout E8.5 to E10.5 (Fig. 3C).

### Defective Yolk Sac Vasculature in Homozygous asTFKI Embryos

In order to address the question whether asTF supported thrombin generation sufficiently to maintain integrity of the yolk sac vasculature, we analyzed procoagulant activity in whole E9.5 embryo lysates in a plasma clotting assay. Procoagulant activity of wt embryos was equivalent to 758±35 pmol recombinant TF/g of total protein. In contrast, asTFKI embryo extracts showed >5 orders of magnitude lower procoagulant activity (p<0.001) (Fig. 3D) and did not shorten clotting times relative to buffer control. Thus, murine asTF expressed in vivo in the absence of flTF lacked measurable procoagulant activity.

**Figure 4:**
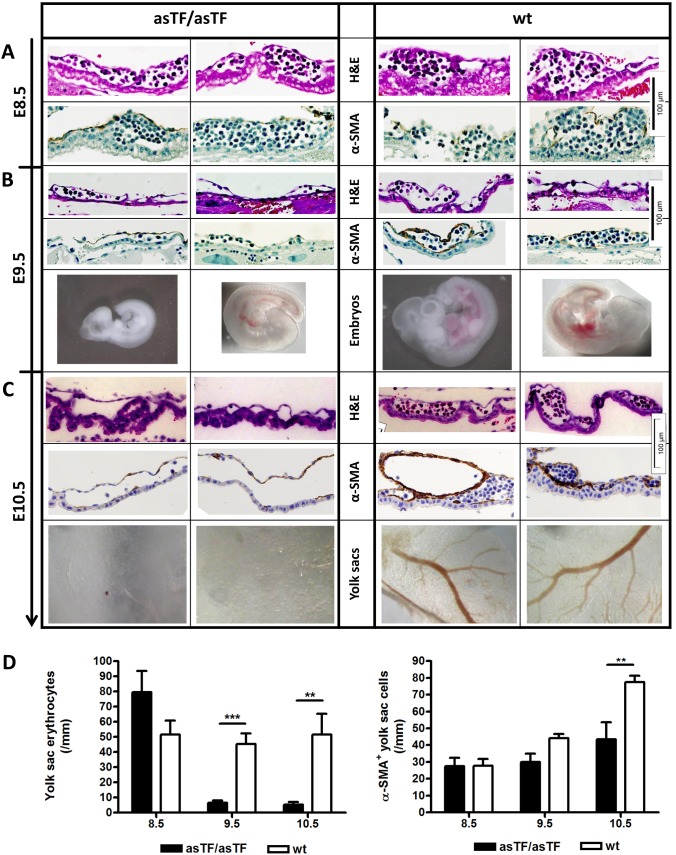
Yolk sac histology. (A) On day E8.5, Haematoxylin and Eosin (H&E) staining and α-SMA immunohistochemistry show comparable integrity, erythrocyte filling, α-SMA staining of the mesodermal layer of homozygous asTFKI and wt yolk sacs. (B) On day E9.5, erythrocyte content, wall thickness, and number of α-SMA expressing cells are reduced in homozygous asTFKI yolk sacs. AsTFKI embryos are found at different stages of growth retardation and wasting. (C) On day E10.5, depletion of erythrocytes, reduced number of α-SMA^+^ cells, thinning and detachment of mesodermal from endodermal layer in homozygous asTFKI yolk sacs. No intact blood filled vessels can be detected anymore in homozygous asTFKI yolk sac en face preparations. (D) Quantification of erythrocyte and α-SMA^+^ cell number in E8.5–E10.5 yolk sacs of homozygous asTFKI and wt embryos ***p<0.001, **p<0.01.

We asked whether asTF supported vessel stability via non-coagulant mechanisms by analyzing yolk sac histology from days E8.5 to E10.5. In homozygous asTFKI mice, early yolk sac vessels developed normally up to day E8.5, were filled with nucleated embryonic erythrocytes, and stained for α-smooth-muscle-actin (α-SMA) in the mesodermal layer, as seen in wt vessels (Fig. 4A). On day 9.5, homozygous asTFKI embryos showed various stages of growth retardation and wasting. Erythrocyte number was reduced (p<0.001); walls of homozygous asTFKI yolk sac vessels appeared thinner, and number of α-SMA expressing cells tended to be reduced (p>0.05, ANOVA; p = 0.07, t-test) compared to yolk sac vessels from wt embryos (Fig. 4B and D). On day 10.5, all homozygous asTFKI embryos were dead and their yolk sac vessels lacked erythrocytes (p<0.01). While yolk sacs of wt embryos had developed large vitelline vessels with high number of α-SMA^+^-cells, homozygous asTFKI yolk sacs displayed thin walled capillaries with low number of α-SMA expressing cells (p<0.01) and partial separation of mesodermal from endodermal layer. En face preparation clearly showed the absence of blood-filled yolk sac vessels in homozygous asTFKI mice (Fig. 4C).

## Discussion

Lethality of TF deficient embryos is associated with a failure to sustain functional yolk sac vasculature [7], [16], [17]. A similar phenotype of the yolk sac vasculature was observed when other clotting factors (FV, prothrombin, and thrombin receptor PAR1) were knocked out, but penetrance and lethality was only 50–60% in the latter [10], [11], [12]. In contrast, deficiency in PAR2, which is activated by the TF/FVIIa complex, did not result in yolk sac abnormalities [18]. In mouse models, human flTF protein levels as low as 1% were sufficient to rescue yolk sac vessel development and preserve survival [8]. Hence, thrombin generation and PAR1 signaling, but not PAR2 signaling, are crucial, but this pathway is not necessarily the only contributor by which TF provides yolk sac vessel integrity.

Additional non-hemostatic effects of both flTF and asTF have been described in tumor angiogenesis [13], [19], [20], [21]. While flTF induces endothelial cell migration via PAR2 [22], asTF acts through direct binding to endothelial integrins [13]. Although vasculogenesis in the early yolk sac is initiated by mesodermal precursor cells forming the primary capillary plexus, migration and differentiation of pericytes/smooth muscles cells is required for stabilization and maturation of the vessels similar to postnatal angiogenesis [23], [24]. The loss of vascular integrity in TF deficient yolk sacs has been attributed to paucity of pericytes [7]. However, it was not known whether asTF might stabilize yolk sac vessels and thus partially rescue total TF deficiency, leading to reduced, 50–60% lethality as seen in knockouts of the thrombin/PAR-1 pathway.

With the newly generated asTFKI mice that express asTF under the control of the endogenous TF promoter, we provide answers to these questions. RNA expression of asTF was 5.9-fold higher in homozygous asTFKI embryos compared to wt embryos, which is consistent with a switch from an allele coding for two splice variants to an allele coding for a single splice variant. The increase in asTF RNA in asTFKI embryos did, however, not fully translate into increased asTF protein expression but yielded asTF protein levels comparable to those of wt embryos. Translation of asTF mRNA in asTFKI embryos might be influenced by the lack of introns in the asTFKI construct, since splicing factors regulate nuclear export and translation of mRNA [25], [26]. AsTF protein expression was analysed using a polyclonal anti-mouse TF antibody [14] and a polyclonal anti-mouse asTF antibody [2]. Both antibodies detected a protein of the same electrophoretic mobility in homozygous asTFKI embryos, which strongly suggests – but does not unequivocally prove – expression of asTF protein. Quantity of material and sensitivity of the antibodies are a limitation in the analysis of asTF protein expression in this study.

Homozygous asTFKI embryos died on day 9.5–10.5. They exhibited yolk sac phenotype similar to the one observed in TF deficient mice [7] with failure of the yolk sac vasculature. Although asTF was expressed at physiologic levels in E9.5 homozygous asTFKI embryos, no pro-coagulant activity could be detected in a sensitive clotting assay. Thus, asTF has no appreciable procoagulant activity in the absence of flTF during embryonic development in vivo. Furthermore, histological analyses suggest that non-hemostatic properties of asTF are insufficient to stabilize yolk sac vessels and assure vascular integrity. Consistently, asTF did not affect pericyte migration, although it increased endothelial cell migration 4–10 fold [13]. Tumors formed by asTF overexpressing flTF-null cells not only displayed high vascularization, but also abundant vascular leakage, further indicating insufficient vessel maturation [20]; on the other hand, asTF potentiated development of functional vessels in vivo when overexpressed in flTF-positive pancreatic cancer cells [27]. These data support the emerging concept that integrin-mediated signaling exerted by asTF, and coagulation-protease signaling by flTF play distinct and non-overlapping roles in post-natal and developmental angiogenesis.
